# Procyanidin B2 alleviates liver injury caused by cold stimulation through Sonic hedgehog signalling and autophagy

**DOI:** 10.1111/jcmm.16733

**Published:** 2021-06-21

**Authors:** Li Ma, Chengxu Li, Shuai Lian, Bin Xu, Hongming Lv, Yanzhi Liu, Jingjing Lu, Hong Ji, Shize Li, Jingru Guo, Huanmin Yang

**Affiliations:** ^1^ College of Animal Science and Veterinary Medicine Heilongjiang Bayi Agricultural University Daqing China

**Keywords:** autophagy, cold stimulation, liver injury, procyanidin B2, sonic hedgehog

## Abstract

Procyanidin B2 (PB2), a naturally occurring flavonoid abundant in a wide range of fruits, has been shown to exert antioxidant, anti‐inflammatory and anticancer properties. However, the role of PB2 in the prevention of cold stimulation (CS)‐induced liver injury. The present study was undertaken to determine the effects of PB2 on liver injury induced by cold stimulation and its potential molecular mechanisms. The present study results showed that treatment with PB2 significantly reduced CS‐induced liver injury by alleviating histopathological changes and serum levels of alanine transaminase and aspartate transaminase. Moreover, treatment with PB2 inhibited secretion of inflammatory cytokines and oxidative stress in cold‐stimulated mice. PB2 reduced cold stimulation‐induced inflammation by inhibiting TLR4/NF‐κB and Txnip/NLRP3 signalling. Treatment with PB2 reduced oxidative stress by activating Nrf‐2/Keap1, AMPK/GSK3β signalling pathways and autophagy. Furthermore, simultaneous application of Shh pathway inhibitor cyclopamine proved that PB2 targets the Hh pathway. More importantly, co‐treatment with PB2 and cyclopamine showed better efficacy than monotherapy. In conclusion, our findings provide new evidence that PB2 has protective potential against CS‐induced liver injury, which might be closely linked to the inhibition of Shh signalling pathway.

## 1 INTRODUCTION

Stress was defined as the adaptive response process to the internal and external or stressors (such as cold, trauma, hypoxia and infection) by the biological organism. Cold stimulation (CS), the main environmental stress that produces reactive oxygen species (ROS), leading to the imbalance of the antioxidant defence system in mammalian cold stress.^1^ Cold weather is a global challenge to human health.^2^ The relationship between extreme cold weather and cardiovascular disease incidence and mortality is obvious. The cold climate is particularly detrimental to the incidence and death of cardiovascular diseases in the elderly.^3^ Many clinical and epidemiological studies have confirmed that cold exposure is related to insufficient cardiovascular response, which may lead to myocardial infarction and arrhythmia.^4^ Previous studies have reported that in addition to causing damage to the heart tissue, acute exposure to cold stress can cause lipid consumption in the adrenal glands and consumption of glycogen in the liver,^5^, ^6^ indicating sympathetic nerve activity and oxidation stress is the source of this fact. In addition, the rate of oxygen consumption increases during cold stress in mammals. Oxidative stress caused by CS results in the accumulation of oxygen free radicals.^7^, ^8^ Under CS conditions, liver tissue is the first tissue to show abnormalities in the body heat production or response.^9^ More importantly, most liver injury caused by CS is accompanied by inflammatory response and oxidative stress. Thus, anti‐inflammation and anti‐oxidative stress may alleviate the potential preventive measures of liver damage caused by CS.

Cold stress affects many cellular processes, leading to physiological and immune responses.^10^ There is evidence that cold stress can trigger inflammatory responses, releasing a large number of inflammatory cytokines.^11^ Inflammatory responses lead to the activation of nuclear factor (NF)‐κB, an inducible transcription factor expressed mainly in lymphocytes and it activates pro‐inflammatory factors. It has been found that cold stress increases mRNA expression of NF‐κB and TNF‐α in quails.^12^ Additionally, oxidative stress can accelerate inflammation by activating pro‐inflammatory pathways, notably NOD‐like receptor protein (NLRP) 3 inflammasome pathway. NLRP3 adaptor is composed of ASC and caspase‐1 and simultaneously induces IL‐1β as a major pro‐inflammatory cytokine, which affects almost every cell type and mediates inflammation in multiple tissues.^13^, ^14^ It is well known that cold stress is closely associated with oxidative stress, leading to excessive accumulation of ROS.^15^, ^16^, ^17^ In addition, as an important antioxidant transcription factor, nuclear factor (erythroid‐derived 2)‐related factor 2 (Nrf2) plays a protective role by regulating the expression of antioxidant proteins to resist oxidative damage.^18^ The target genes of Nrf2 are involved in synthesizing of GSH and eliminating ROS, and Kelch‐like ECH‐associated protein 1 (Keap1) is essential for regulating the activity of Nrf2.^19^, ^20^ Under normal circumstances, Nrf2 is continuously degraded in a Keap1‐dependent manner by the ubiquitin‐proteasome pathway. The degradation of Nrf2 can be suspended in the presence of ROS, and stable Nrf2 accumulates in the nucleus and activates the target genes for cell protection against oxidative stress.^21^, ^22^ More importantly, autophagy is an intracellular pathway through which lysosomes degrade and recover proteins and organelles. Lysosomes are generally considered one of the main targets of ROS.^22^ Recent reports indicate that autophagy is widely regarded as a key regulator of cell survival and homeostasis, and the lack of autophagy promotes inflammatory response and oxidative stress.^23^, ^24^ In particular, previous studies have shown that autophagy disorders can also exacerbate liver disease. Dihydromyricetin regulates autophagy through the Nrf2 and p62 signalling pathway and thereby reducing ethanol‐induced liver damage.^25^ Recent study also shown that autophagy has a crucial role in the regulation of non‐alcoholic fatty liver disease.^26^ Macrophage autophagy prevents liver fibrosis in mice.^27^ Recent studies indicate the important role of autophagy and oxidative stress, inflammatory responses in various liver diseases. However, their exact role in liver injury induced by CS has not been fully elucidated.

Hedgehog signalling is essential for development during embryogenesis, regulating the wound healing response of adult tissues and homeostasis. When ligands such as Sonic Hedgehog (SHH) bind to Patched (Ptc) receptors, this pathway is activated, leading to the release of Ptc‐mediated smoothing (Smo) inhibition.^28^, ^29^ Subsequently, Smo induced the activation and nuclear accumulation of Gli transcription factors, which in turn triggered the activation of a large number of target genes.^30^, ^31^ According to reports, Shh signalling is abnormally activated in various liver pathological conditions (such as inflammation, liver regeneration, liver fibrosis and HCC).^32^, ^33^, ^34^, ^35^ However, the mechanism of Shh signalling pathway in CS‐induced liver injury has not been elucidated.

Procyanidins are a subtype of flavonoid family that possess abundant biological functions, including anti‐inflammatory, antitumor and antioxidant activities.^36^, ^37^, ^38^ Procyanidin B2 (PB2), a B‐type dimer of procyanidin, has been shown to possess greater antioxidant and anti‐inflammatory effects than other procyanidins. A previous study has shown that PB2 as the ingredient in pericarp extract of *Annona crassiflora* which exhibits hepatoprotective properties.^39^ The protective mechanism of PB2 in acute liver damage induced by CCl_4_ was closely related to inhibiting inflammatory response and apoptosis.^40^ Additionally, procyanidin could trigger autophagy of human hepatoma G2 cells via ROS generation.^41^ However, the protective effect of PB2 on CS‐induced liver injury has not been reported. Therefore, the present study was undertaken to investigate the effects of PB2 on CS‐induced liver injury and the potential mechanisms, with particular attention to the activation of autophagy and association with the Shh signalling pathway.

## 2 MATERIALS AND METHODS

All experimental procedures were performed in accordance with the Guidelines established in Heilongjiang Bayi Agricultural University (Daqing, China) for the Care and Use of Experimental Animals. The Animal Ethics Committee of Heilongjiang Bayi Agricultural University approved the study protocol.

### 2.1 Reagents

Procyanidin B2 (PB2), 98% pure, was from Herbpurify (CAS NO: 29106‐49‐8). Dimethyl sulphoxide (CAS NO: 67‐68‐5) and d‐Galactosamine (CAS NO: 1772‐03‐8) were obtained from Sigma Chem. Co. Cyclopamine (CAS NO: HY‐17024) was obtained from MCE. Co. Antibodies against NFE2L2 (1:1000; cat. no. Ab89443), Keap1 (1:1000; cat. no. Ab118285), AMP‐activated protein kinase (AMPK; 1 μg/mL; cat.no. Ab80039), phosphorylated AMPK (p‐AMPK; 1:5000; cat.no. Ab133448), phosphorylated GSK3β (p‐GSK3β; 1:10 000; cat.no. Ab75814) and phosphorylated mTOR (p‐mTOR, phosphorylation site Ser2448; 1:5000; cat.no. Ab109268) were from Abcam. Caspase‐1 (1:1000; cat. no. #24232), cleaved‐IL‐1β (1:1000; cat. no. #63124), Phospho‐NF‐κB p65 (1:1000; cat. no. #3033) and phosphorylation‐IκBα (p‐IκBα; 1:1000; cat. no. #4814) were obtained from Cell Signaling Technology, Inc. SOD1 (1:2000; cat. no. 10269‐1‐AP), HO‐1 (1:2000; cat. no. 27282‐1‐AP), Catalase (1:2000; cat. no. 21260‐1‐AP), TLR4 (1:1000; cat. no. 19811‐1‐AP), GSK3β (1:2000; cat. no. 22104‐1‐AP), Beclin1 (1:2000; cat. no. 11306‐1‐AP), LC3 (1:2000; cat. no. 14600‐1‐AP), NLRP3 (1:1000; cat. no. 19771‐1‐AP), ASC (1:2000; cat. no. 10500‐1‐AP), IL‐1β (1:2000; cat. no. 16806‐1‐AP), p65 (1:2000; cat. no. 66535‐1‐Ig), IκBα (1:2000; cat. no. 10268‐1‐AP), PI3K (1:1000; cat. no. 20584‐1‐AP), p‐AKT (phosphorylation site Ser473)(1:2000; cat. no. 66444‐1‐Ig), AKT (1:1000; cat. no. 10176‐2‐AP), mTOR (1:500; cat. no. 20657‐1‐AP), Shh (1:1000; cat. no. 20697‐1‐AP), Smo (1:2000; cat. no. 66851‐1‐Ig), Gli1 (1:2000; cat. no. 66905‐1‐Ig) and β‐actin (1:10 000; cat. no. 66009‐1‐Ig) were obtained from Proteintech. Additionally, alanine aminotransferase (ALT cat. no. C009‐2‐1), aspartate aminotransferase (AST cat. no. C010‐2‐1), Reactive Oxygen Species (ROS) Assay Kit (cat. no. E004‐1‐1) and hydroxyproline detection kits were purchased from Nanjing Jiancheng Bioengineering Institute. Superoxide dismutase (SOD), glutathione peroxidase (GSH‐Px) and malondialdehyde (MDA) test kits were obtain Beyotime Institute (Shanghai, China). All other reagents were purchased from Sigma‐Aldrich, if not otherwise indicated.

### 2.2 Animal model of cold stimulated‐induced liver injury

C57BL/6 male mice weighing 22‐24 g, 5 weeks old, were purchased from Charles River Lab and raised under environmentally controlled conditions (temperature 24℃ ± 2℃, humidity 40% and a 12‐hours light/dark cycle) with food and sterile water ad libitum for 1 week. After acclimatization, the cold‐stimulated (CS) group were transferred to a 4℃ climatic chamber and kept for 4 hours per day, and transferred back to room temperature by day (8:00 AM to 8:00 PM). The process of chronic CS persists for 3 weeks.^42^ Mice were randomly divided into seven groups (n = 6 mice per group): Control group, no treatment; Cold‐Stimulated group (CS group); PB2 (50 mg/kg or 100 mg/kg, iG) group; PB2 (50 mg/kg or 100 mg/kg bw per day, iG) + CS group.

To further verify the effect of PB2 on Hh pathway, mice were treated with Hh inhibitor cyclopamine intragastrically (iG). Mice were randomly divided into five groups (n = 6 mice per group): CS group; PB2 (100 mg/kg bw per day, iG) group; Cyclopamine (20 mg/kg bw per day, iG) group; PB2 (100 mg/kg bw per day, iG) + Cyclopamine (20 mg/kg bw per day, iG) group; Cyclopamine (20 mg/kg bw per day, iG) + CS group; PB2 (100 mg/kg bw per day, iG) + Cyclopamine (20 mg/kg bw per day, iG) + CS group. After 24 hours of the last treatment, all mice were anaesthetized with pentobarbital intraperitoneally. Subsequently, liver tissue and serum were collected and used for biomarker profiling, histopathological analysis, ELISA or Western blot assays.

### 2.3 Histopathological evaluation and Immunohistochemistry (IHC) staining

Formalin‐fixed, paraffin‐embedded liver tissue sample was cut into 5 μm thick sections and then stained with haematoxylin‐eosin and liver IHC staining for Shh, Smo and Gli1 to evaluate liver pathological lesions under light microscopy.

### 2.4 Measurement of liver function index and oxidative stress

All mice were killed the last cold stimulation treatment, and liver and blood were collected for biochemical analysis. ALT and AST levels in serum and liver were measured using the corresponding detection kits in accordance with the manufacturer's instructions. All mice were killed in the final cold stimulation treatment, and liver and blood were collected for biochemical analysis. According to the manufacturer's instructions, use the corresponding test kit to measure the ALT and AST levels in the serum and liver. In addition, the mouse liver tissue was homogenized and dissolved in extraction buffer, and its concentration was normalized and then subjected to superoxide dismutase (SOD), glutathione peroxidase (GSH‐Px) and malondialdehyde (MDA) assays, such as described in the Total Superoxide Dismutase Assay Kit with nitroblue tetrazolium (catalog no. S0109), Total Glutathione Peroxidase Assay Kit (catalog no. S0058) and Lipid Peroxidation MDA Assay Kit (catalog no. S0131S), respectively (Beyotime Institute of Biotechnology, Jiangsu, China). The concentrations of SOD, GSH‐Px and MDA were detected using multimode microplate readers (Multiskan FC, Thermo Fisher Scientific) at 560, 340 and 530 nm, respectively. Furthermore, the ROS levels in liver tissues and cells were determined using Reactive Oxygen Species Assay Kit (catalog no. E004‐1‐1, NanJing JianCheng Bioengineering Institute, Jiangsu, China) according to the manufacturer's protocols and detected using multimode microplate readers at 520 nm. In addition, mice liver tissues were homogenized and dissolved in extraction buffer to analyse the MDA, ROS, SOD and GSH levels according to the manufacturer's instructions. All results were normalized by the total protein concentration in each sample.

### 2.5 Enzyme‐linked immunosorbent assay (ELISA)

Blood serum was collected for measurement of the inflammation biomarkers TNF‐α, IL‐6 and IL‐1β levels by ELISA kits as the manufacturer's instructions (BioLegend), and the absorbance at 450 nm was read.

### 2.6 Western blot analysis

Western blot was performed as previously described by Xu et al.^43^ In brief, total protein was extracted from the liver tissues using a protein extract kit according to the manufacturer's protocol. All protein concentrations were measured with BCA protein assay. A total of 20 μg of protein from each sample were separated by a 12% SDS‐PAGE and transferred onto 0.45 μm polyvinylidene difluoride (PVDF) membrane. The membrane was blocked with 5% (w/v) non‐fat milk for 2 hours followed by incubated with primary antibodies (Nrf2, Keap1, AMPK, GSK3β, Beclin1, LC3, NLRP3, ASC, Casapase‐1, IL‐1β, p‐p65/p65, p‐IκBα/IκBα, Shh, Smo, Gli1 and β‐actin) and secondary antibody (anti‐rabbit IgG and anti‐mouse IgG). Lastly, the membranes were visualized with the enhanced chemiluminescence (ECL) reagent in Western blotting analysis system with Image Lab (Bio‐Rad).

### 2.7 Statistical analysis

All data were analysed using the appropriate statistical analysis methods with the Statistical Package for the SPSS software version 25.0 (IBM) and GraphPad Prism program (Prism 8.3.0; GraphPad Software). All data were tested for normality and homogeneity of variance using the Shapiro‐Wilk and Levene tests, respectively. One‐way ANOVA was performed for multiple comparisons with Bonferroni correction. A *P*‐value <0.05 was considered statistically significant, and a *P*‐value <0.01 was considered highly significant.

## 3 RESULTS

### 3.1 PB2 relieves CS‐induced liver injury

**FIGURE 1:**
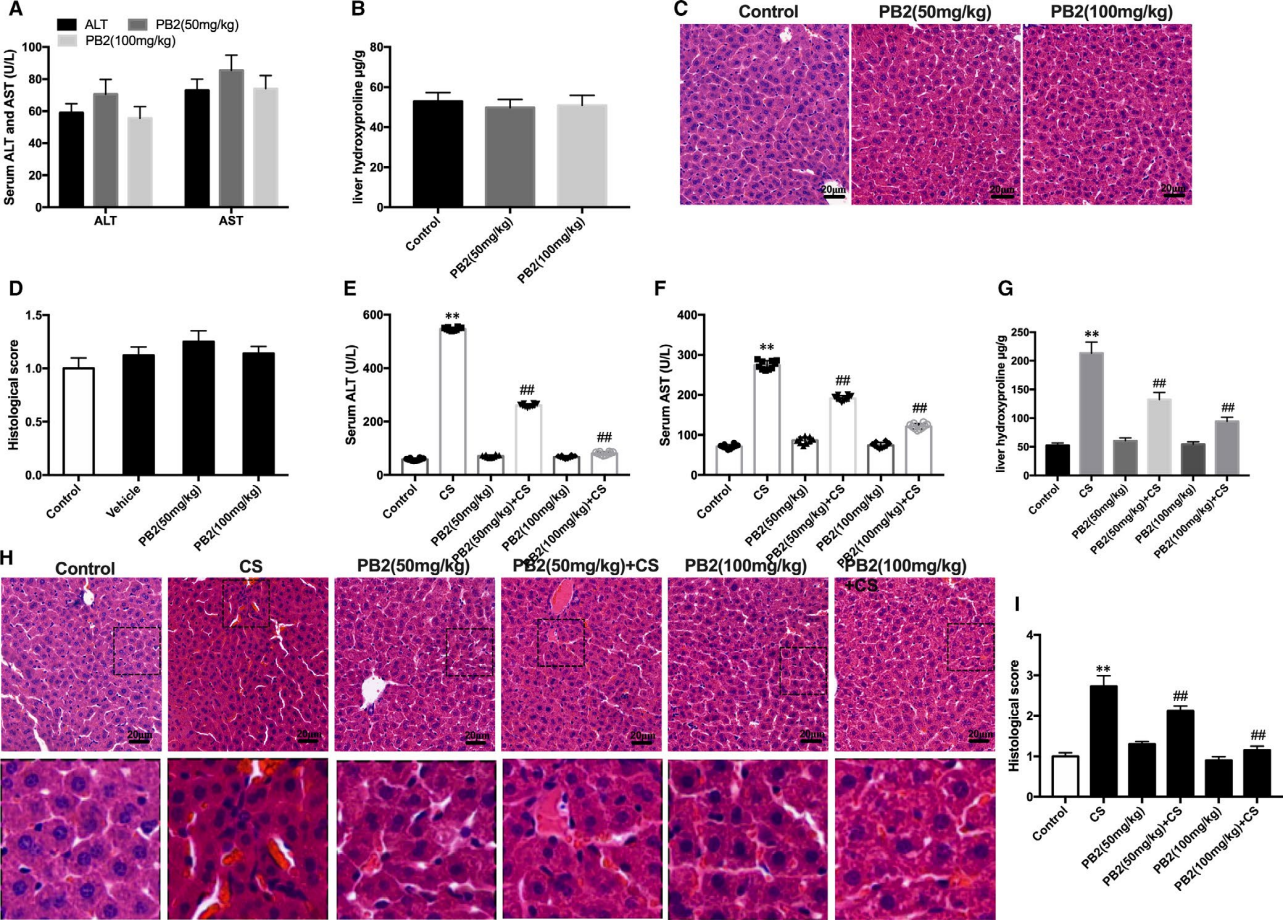
PB2 relieved CS‐induced liver injury in mice. A, B, Serum ALT and AST levels and liver hydroxyproline concentrations. Data are presented as the mean ± SD (n = 10, from three independent experiments). C, D, The H&E staining of liver tissues in the Control, vehicle and PB2 groups (original magnification, 200×). The structures were clear, and the hepatocytes were well‐preserved with clear cytoplasm and prominent nuclei and nucleoli. E‐G, Detection of ALT, AST and liver hydroxyproline concentrations in serum of PB2‐treated mice after cold stimulation. H, I, H&E staining of liver tissues, Scale bar represents 20 μm (original magnification, 200×). Similar results were obtained from three independent experiments. All data are presented as the mean ± SEM (n = 6 in each group). *P < 0.05 and **P < 0.01 vs Control group; ^##^ *P* < 0.01 vs CS group

To investigate whether PB2 alleviated CS‐induced liver injury in mice, the effect of various dose of PB2 on liver safety was measured. Compared with the control group, treatment with vehicle and PB2 (50 and 100 mg/kg) have no significant effect on serum activities of ALT, AST or the hydroxyproline concentration of the liver tissue (Figure 1A,B). Histological analysis of liver slices in the four groups revealed normal morphology (Figure 1C,D). These results clearly demonstrated that treatment with PB2 at 50 and 100 mg/kg had no effect on liver toxicity in mice.

Cold stimulation significantly up‐regulated serum ALT, AST and liver hydroxyproline levels, whereas PB2 treatment significantly reduced serum activities of ALT, AST and liver hydroxyproline levels in a dose‐dependent manner (Figure 1E‐G). Histological analysis showed that liver tissue was well structured and hepatocytes were with clear cytoplasm and prominent nuclei in the control group. However, significant structural disturbances such as bleeding, neutrophil infiltration and hepatocyte necrosis were observed in CS group, whereas PB2 treatment alleviated the pathological changes induced by CS (Figure 1H,I). These results clearly demonstrated that the hepatoprotective effect of PB2 treatment on CS‐induced liver injury was dose‐dependent.

### 3.2 PB2 suppressed inflammatory responses in CS‐induced liver injury

**FIGURE 2:**
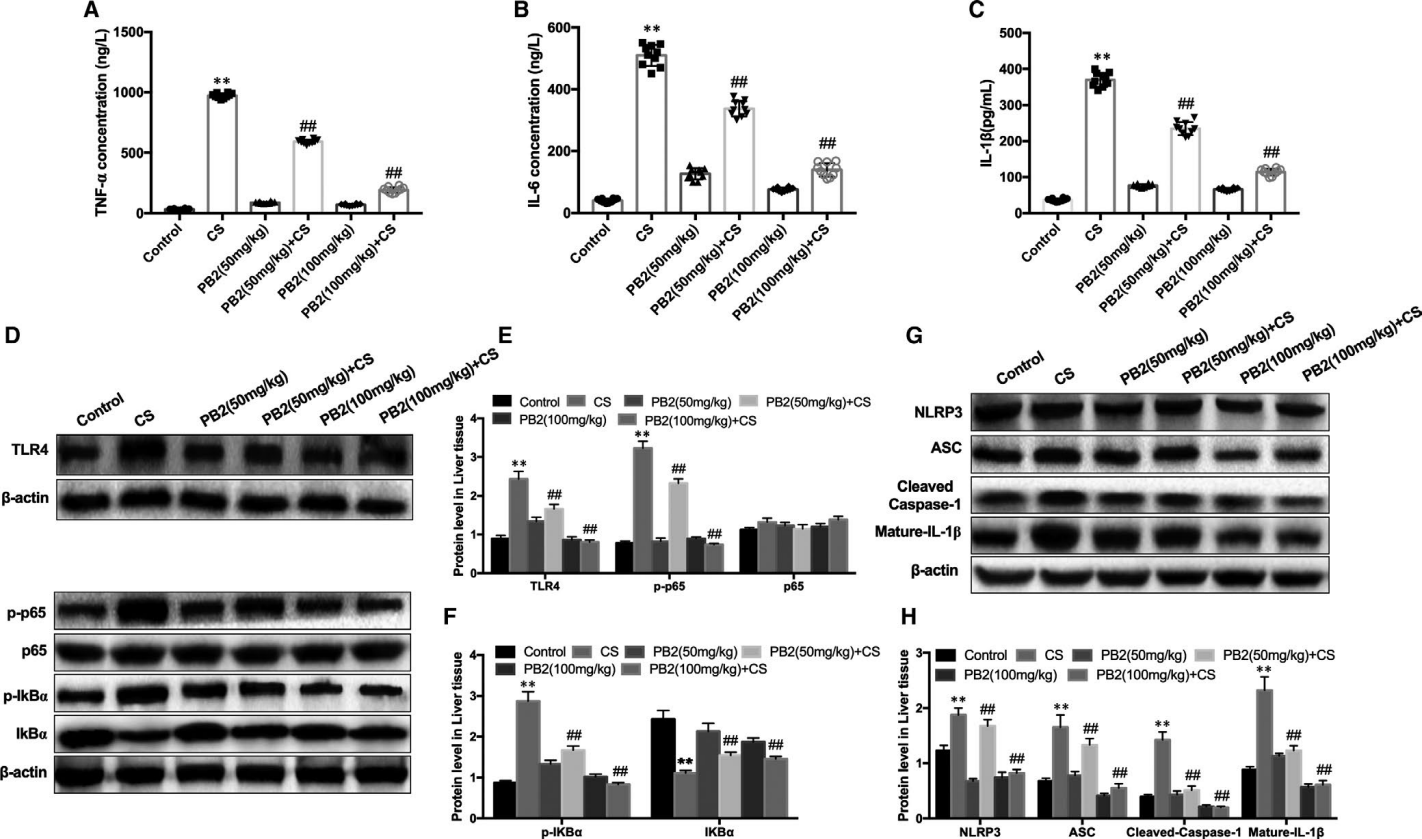
PB2 treatment suppressed CS‐activated inflammatory response of liver injury in mice. A‐C, Effects of PB2 on CS‐induced serum TNF‐α, IL‐6 and IL‐1β generation. Similar results were obtained from three independent experiments. D‐F, Effects of PB2 on p‐p65, p65, p‐IκBα, p‐IκBα and TLR4 protein expression were measured by Western blotting, and quantification of protein expression was performed by densitometric analysis. G‐H, Effects of PB2 on NLRP3, ASC, cleaved‐caspase‐1 and mature‐IL‐1β protein expression were measured by Western blotting, and quantification of protein expression was performed by densitometric analysis. Similar results were obtained from three independent experiments. All data are presented as the mean ± SEM (n = 6 in each group). *P < 0.05 and **P < 0.01 vs Control group; ^##^ *P* < 0.01 vs CS group

Expression of inflammatory factors TNF‐α, IL‐6 and IL‐1β is related to liver injury. The levels of inflammatory cytokines in serum induced by CS were measured using ELISA. Cold stimulation significantly stimulated secretion of TNF‐α, IL‐6 and IL‐1β in the serum compared to the control group, whereas PB2 treatment reduced the production of inflammatory cytokines induced by CS (Figure 2A‐C). The TLR4 signalling pathway is considered a key pathway that mediates inflammatory responses, and it functions upstream of NF‐κB. Therefore, we examined the effect of PB2 on TLR4/NF‐κB signalling pathways under cold stimulation. The results showed that compared to the CS group, PB2 significantly reduced phosphorylation of NF‐κB (p65), prevented phosphorylation and degradation of IκBα and inhibited up‐regulation of TLR4 expression (Figure 2D‐F). These results demonstrate that inflammatory responses attenuated by PB2 may partly contribute to suppression of the TLR4/NF‐κB signalling pathway.

### 3.3 PB2 treatment suppressed Txnip/NLRP3 inflammasome signalling pathway in mice with CS‐induced liver injury

We investigated whether liver damage caused by CS also triggered activation of NLRP3 inflammation. Western blotting showed that cold stimulation significantly increased abundance of NLRP3, ASC, cleaved‐caspase‐1 (p20) and mature‐IL‐1β (p17) proteins. PB2 treatment dramatically inhibited expression of NLRP3, ASC, cleaved‐caspase‐1 and mature‐IL‐1β proteins (Figure 2G,H), suggesting that PB2 inhibits inflammation partly through the inhibition of the NLRP3 inflammasome.

### 3.4 PB2 treatment alleviated oxidative stress in mice with CS‐induced liver injury

**FIGURE 3:**
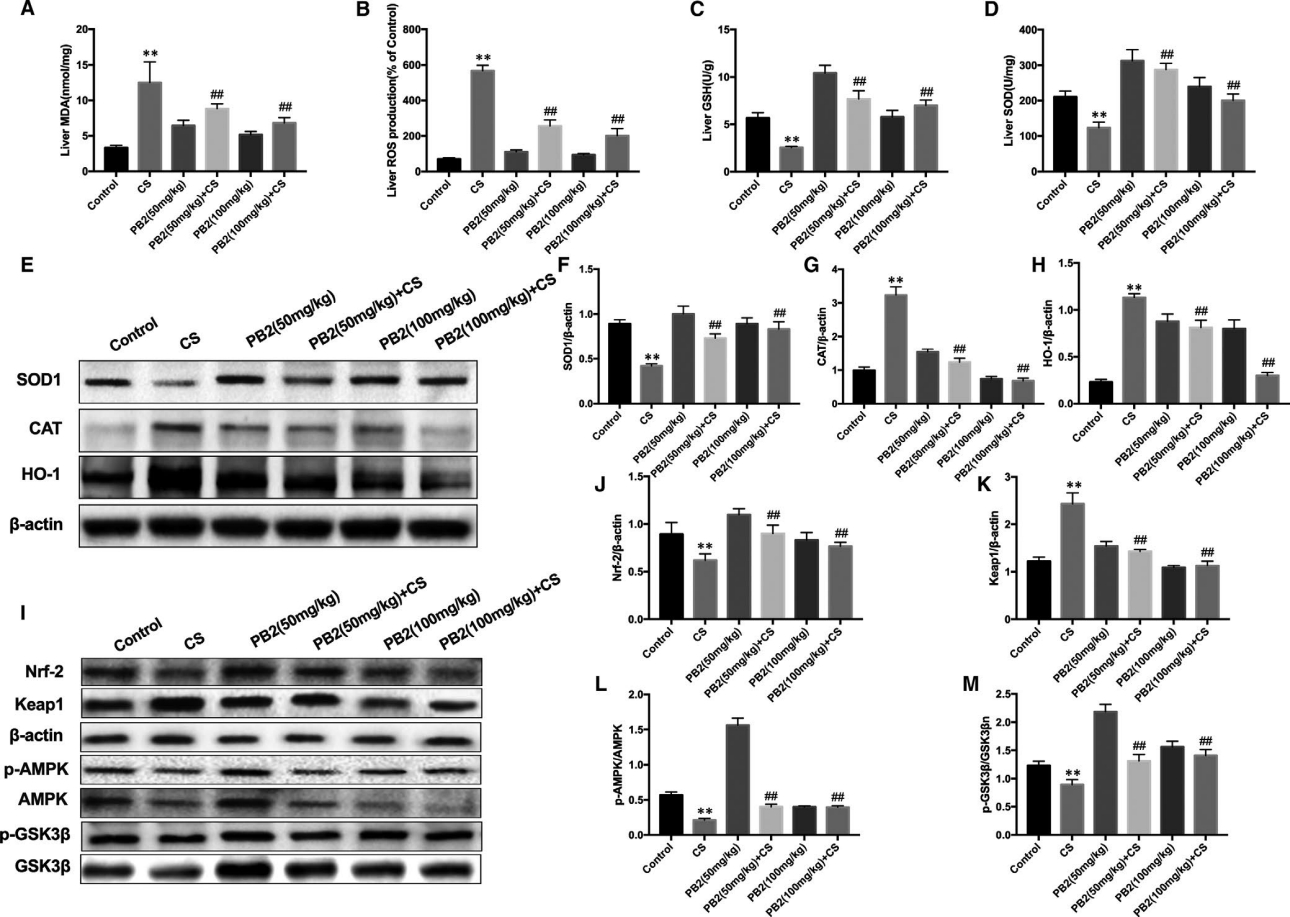
Effects of PB2 on oxidative stress in CS‐induced liver injury in mice. A‐D, Effect of PB2 on levels of ROS, MDA, SOD and GSH. Similar results were obtained from three independent experiments. E, Effects of PB2 on SOD1, CAT and HO‐1 protein expression were measured by Western blotting. F‐H, Quantification of protein expression was performed by densitometric analysis. I, Effects of PB2 on Nrf2, Keap1, p‐AMPK, AMPK, p‐GSK3β and GSK3β protein expression were measured by Western blotting. J‐M, Quantification of protein expression was performed by densitometric analysis. Similar results were obtained from three independent experiments. All data are presented as the mean ± SEM (n = 6 in each group). *P < 0.05 and **P < 0.01 vs Control group; ^##^ *P* < 0.01 vs CS group

Cold stress alters homeostasis, which results in production of ROS and alterations in the antioxidant defence system.^1^ Oxidative damage is also one of the main factors of liver injury in mice caused by CS. Therefore, we measured whether PB2 improved cold‐induced liver oxidative damage. CS increased excess accumulation of MDA and ROS, and consumption of GSH and SOD, leading to oxidative damage in the liver of mice. However, PB2 treatment reversed these effects (Figure 3A‐D). Expression of SOD1, CAT and HO‐1 proteins was consistent with the above results (Figure 3E‐H). To investigate further the protective mechanism of PB2 treatment on CS‐induced liver injury, we analysed involvement of the Nrf2/Keap1 and AMPK/GSK3β signalling pathways by Western blotting. PB2 treatment increased AMPK and GSK3β phosphorylation and enhanced Nrf2 and Keap1 expression compared with the CS group (Figure 3I‐M). These results show that PB2 may protect against oxidative stress damage induced by CS by enhancing the Nrf2/Keap1 and AMPK/GSK3β signalling pathways.

### 3.5 PB2 treatment induced autophagy in mice with CS‐induced liver injury

**FIGURE 4:**
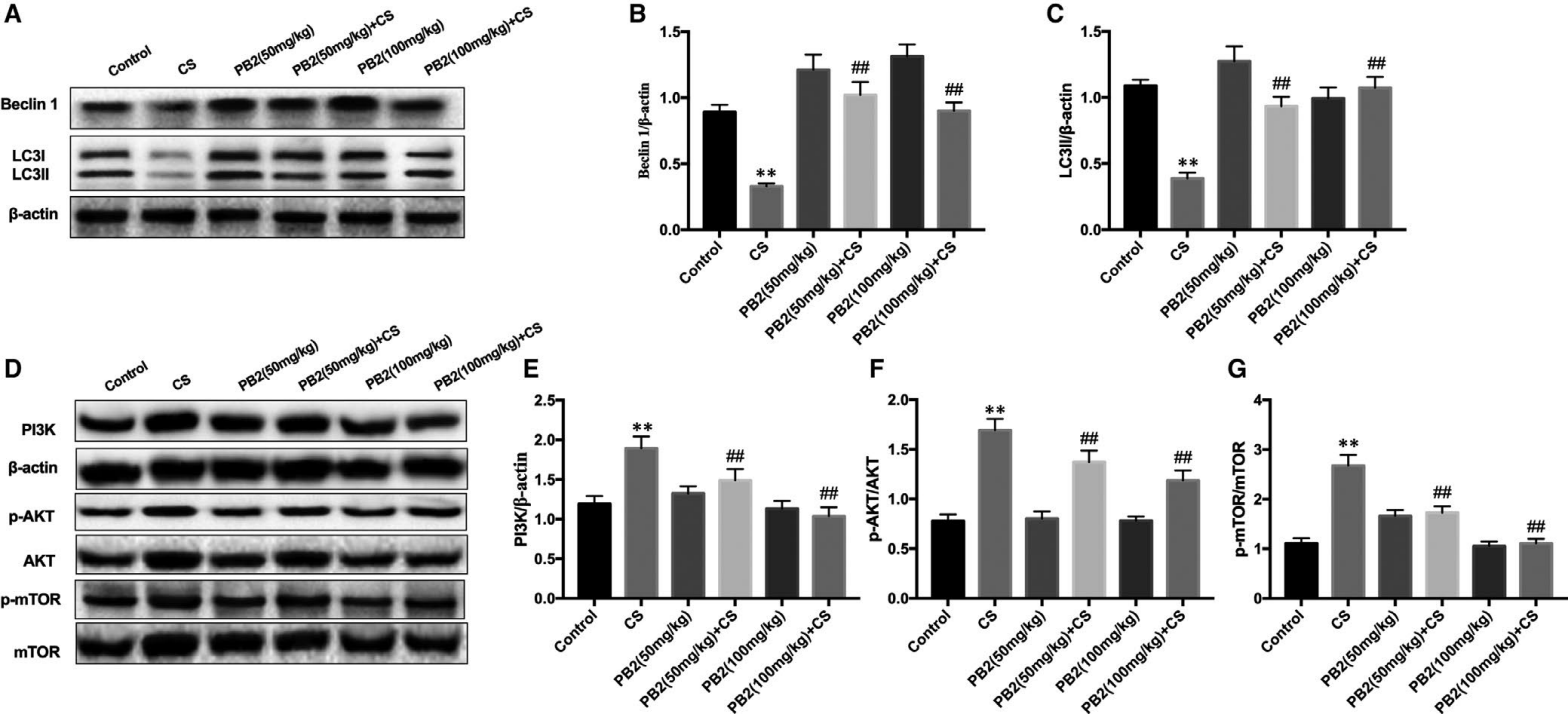
Effect of PB2‐mediated autophagy on liver injury induced by CS in mice. A, Effects of PB2 on Beclin‐1 and LC3 protein expression were measured by Western blotting. B‐C, Quantification of protein expression was performed by densitometric analysis. E, Effects of PB2 on PI3K, p‐AKT, AKT, p‐mTOR and mTOR protein expression were measured by Western blotting. D‐G, Quantification of protein expression was performed by densitometric analysis. Similar results were obtained from three independent experiments. All data are presented as the mean ± SEM (n = 6 in each group). *P < 0.05 and **P < 0.01 vs Control group; ^##^ *P* < 0.01 vs CS group

Autophagy can regulate inflammatory responses and oxidative stress and plays an essential role in the amelioration of liver injury induced by CS. We examined the protein expression of key autophagy genes, including Beclin‐1 and LC3. The expression of key autophagy proteins was decreased by CS but recovered by PB2 treatment (Figure 4A‐C). To investigate further the protective mechanism of PB2 treatment on CS‐induced liver injury, involvement of the PI3K/AKT/mTOR signalling pathway were carried out by Western blotting analysis. PB2 attenuated expression of PI3K, p‐AKT and p‐mTOR, which was conducive to activation of autophagy (Figure 4D‐G). This indicates that PB2‐induced autophagy activation may be dependent on the PI3K/AKT/mTOR signalling pathway.

### 3.6 PB2 alleviates CS‐induced liver injury by cooperating with the Shh pathway and autophagy

**FIGURE 5:**
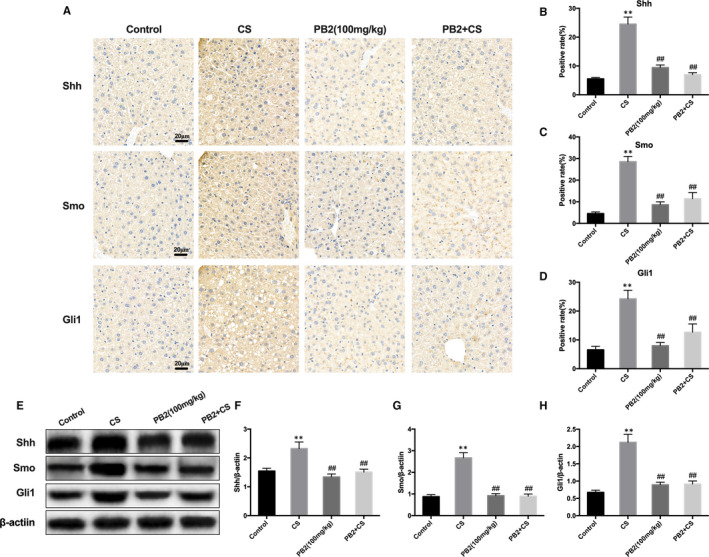
PB2 attenuated increase in Shh signalling pathway induced by CS. A, IHC staining and quantitative analysis of Shh, Smo and Gli1 in liver tissues, and scale bar represents 20 μm. B‐D, Quantitative analysis of Shh, Smo and Gli1 in liver tissues. E, Expression of Shh, Smo and Gli1 protein in liver tissues. F‐H, Quantitative analysis of Western blotting results. Similar results were obtained from three independent experiments. All data are presented as the mean ± SEM (n = 6 in each group). *P < 0.05 and **P < 0.01 vs Control group; ^##^ *P* < 0.01 vs CS group

**FIGURE 6:**
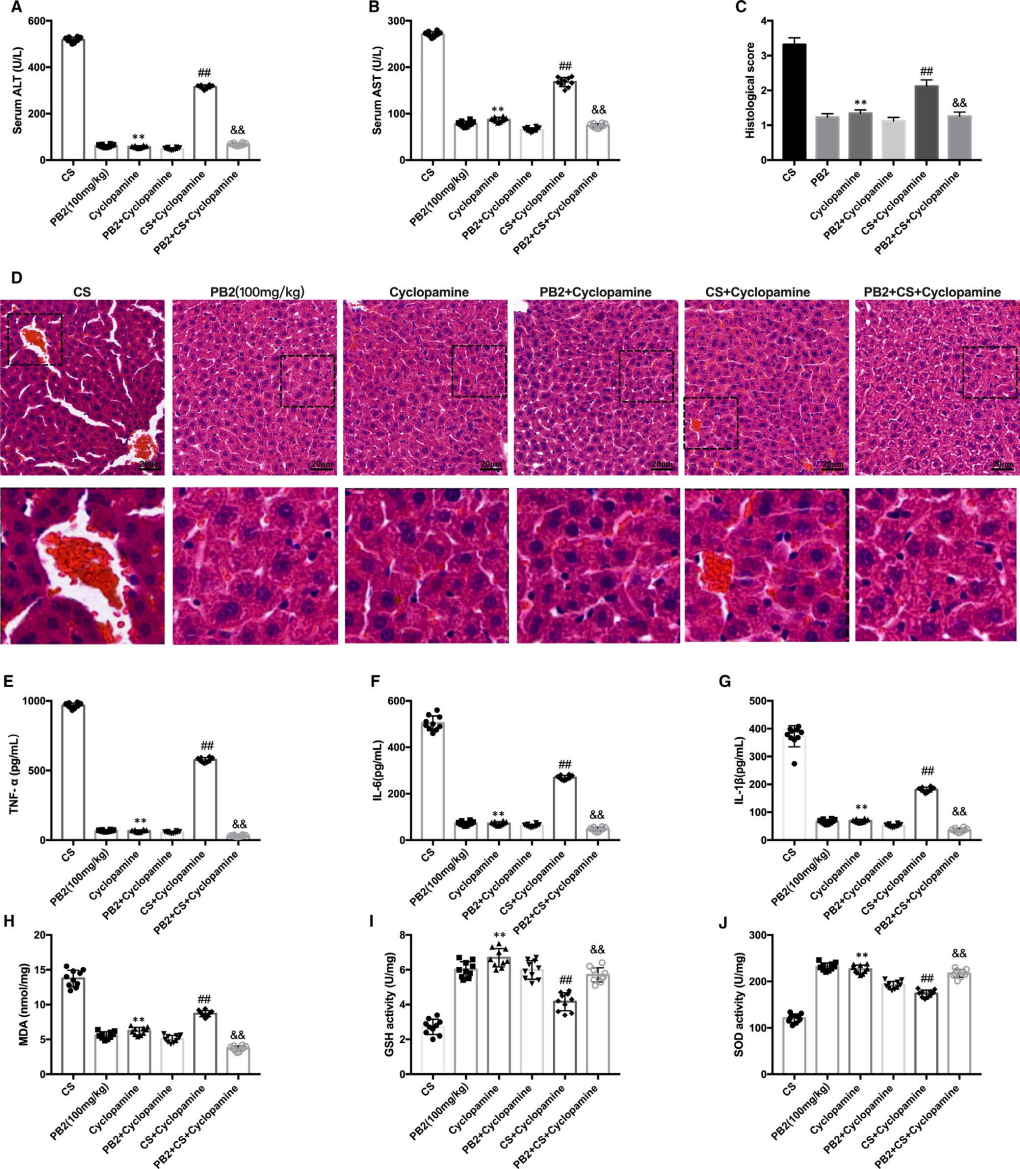
PB2 alleviated CS‐induced liver injury by cooperating with Shh pathway. A, B, Serum concentrations of ALT and AST. Data are presented as the mean ± SD (n = 10, from three independent experiments). C, D, H&E staining of liver tissues, scale bar represents 20 μm (original magnification, 200×). E‐G, Measurement of TNF‐α, IL‐6 and IL‐1β concentrations. H‐J, Measurement of MDA, GSH and SOD concentrations. Similar results were obtained from three independent experiments. All data are presented as the mean ± SEM (n = 6 in each group). *P < 0.05 and **P < 0.01 vs CS group; ^##^ *P* < 0.01 vs CS group; ^&&^ *P* < 0.01 vs PB2+Cyclopamine group

**FIGURE 7:**
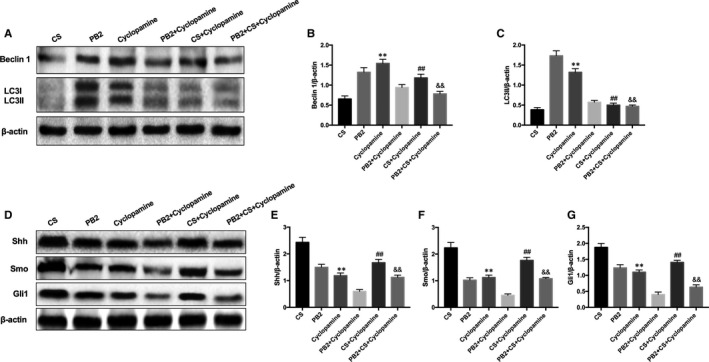
PB2 alleviated CS‐induced liver injury by cooperating with Shh pathway and autophagy. A, Expression of Beclin‐1 and LC3 protein in liver tissues. B‐C, Quantitative analysis of Western blotting results. D, Expression of Shh, Smo and Gli1 protein in liver tissues. E‐G, Quantitative analysis of Western blotting results. Similar results were obtained from three independent experiments. All data are presented as the mean ± SEM (n = 6 in each group). *P < 0.05 and **P < 0.01 vs Control group; ^##^ *P* < 0.01 vs CS group; ^&&^ *P* < 0.01 vs PB2+Cyclopamine group

To determine further the molecular mechanism of PB2 in CS‐induced liver injury, the involvement of the Hh pathway was studied. In mice with CS‐induced liver injury, significant up‐regulation in the expression of Shh, Smo and Gli1 were revealed by IHC staining compared with the control group. PB2 treatment significantly decreased the protein abundance of Shh, Smo and Gli1 (Figure 5A‐D). Western blotting results were consistent with IHC staining (Figure 5E‐H). We speculated whether PB2 played a role in the Shh signalling pathway and autophagy in CS‐induced liver injury. Treatment with PB2 and Hh pathway inhibitor cyclopamine both reduced pathological damage, inflammation and oxidative stress in the liver. Combined treatment with PB2 and cyclopamine showed a more significant relaxation effect than either treatment alone in CS‐induced liver injury (Figure 6). Western blotting of proautophagic proteins Beclin‐1 and LC3 in liver tissue also showed that combined treatment with PB2 and cyclopamine has a more significant effect on activation of autophagy and decreased expression of Shh, Smo and Gli1 (Figure 7). These results indicated that Hh inhibitor cyclopamine reduced liver injury as effectively as PB2, and combined treatment with PB2 and cyclopamine showed a better effect than single treatment.

## 4 DISCUSSION

Liver injury is a major health issue worldwide which can develop to liver fibrosis and even hepatic carcinoma.^44^ During cold stimulation, changes in antioxidant defence systems and anti‐inflammatory responses are activated in the liver.^1^ Therefore, reducing inflammatory response and oxidative stress can prevent or treat liver injury. It has been reported that proanthocyanidins have antioxidant and anti‐inflammatory properties, which may be closely related to the defence mechanism of autophagy. We found that PB2 has a protective effect on CS‐induced liver injury and found for the first time that PB2 has an effective antioxidant and anti‐inflammatory effect on CS‐induced liver injury through a mechanism that depends on Shh signalling and autophagy.

Accumulating evidence suggests that cold stress is the most common source of environmental stress and can damage the nervous, cardiovascular and immune systems.^45^, ^46^ The liver is an important heat‐generating organ in the body, which is responsible for heat production during acute cold stimulation to maintain core body temperature.^47^ Cold‐stress‐induced liver injury significantly increases serum ALT and AST levels and liver histopathological changes. Treatment with PB2 significantly prevented these increases, indicating that PB2 protects liver tissue from cold‐stimulated liver injury. It is well known that cold stimulation causes liver damage by inducing oxidative stress and inflammation.^48^, ^49^ To further investigate the effect of PB2 on inflammatory responses and oxidative stress, serum levels of inflammatory cytokines and liver abundance of oxidative markers were measured. The present study showed that PB2 treatment significantly reduced production of IL‐1β, IL‐6 and TNF‐α, reduced levels of MDA and ROS, and reversed consumption of GSH and SOD. Furthermore, PB2 treatment reversed histological changes induced by CS in mice. These results indicate that PB2 treatment alleviated the hepatic inflammation, oxidative stress and pathological damage in mice.

It is well known that the signalling cascade that produces pro‐inflammatory cytokines is mainly regulated by NF‐κB/TLR4‐mediated signalling.^50^ TLR4 recognizes endogenous ligands induced during the inflammatory response and then activates NF‐κB through phosphorylation and degradation of IκBα, thereby producing inflammatory factors and ultimately causing liver inflammation.^51^ Additionally, activation of NF‐κB is the basic initial step to trigger activation of NLRP3, and ROS produced by NF‐κB‐mediated inflammation.^52^ After activation of NLRP3, the adapter protein ASC is required to activate caspase‐1 further. The maturation of inflammatory cytokine IL‐1β is related to the pathogenesis of liver injury.^53^ Our results indicate that PB2 significantly inhibit the protein expression cold stimulation‐induced NLRP3, ASC, Caspase‐1 cleavage and I mature L‐1β. Moreover, activation of Nrf2 could improve various diseases caused by inflammation and oxidative stress.^54^, ^55^ Under normal circumstances, Nrf2 is continuously degraded in a Keap1‐dependent manner through the ubiquitin‐proteasome pathway.^56^ However, after exposure to stress inducers, the Nrf2 released from Keap1 translocates into the nucleus, heterodimerizes with the small Maf protein and activates cells through antioxidant response elements/electrophilic response elements.^22^ There is increasing evidence that during this process, AMPK leads to the accumulation of Nrf2 nuclear transcription by inhibiting phosphorylation of GSK3β, thereby attenuating stress‐induced liver injury.^57^ AMPK has the ability to maintain metabolic homeostasis and plays a key role in the survival of cells and organisms during metabolic stress. However, it also controls the redox state and mitochondrial function.^58^ AMPK‐related pathways may inhibit oxidative stress and inflammation‐induced liver injury. Our results indicate that PB2 inhibits activation of NF‐κB/TLR4 and NLRP3 inflammasome, whereas simultaneous activation of Nrf2/Keap1 and AMPK/GSK3β signalling pathways may contribute to the antioxidant and anti‐inflammatory effects and thus alleviate CS‐induced liver injury. Based on the above results, we clarified that PB2‐alleviated CS‐induced liver injury may be related to anti‐inflammatory and antioxidant damage.

We speculate that PB2 may mediate other mechanism to predominantly relieve CS‐induced liver injury. A large number of experimental studies have shown that enhancing autophagy can reduced inflammation and improve liver toxicity induced by LPS/GalN.^59^ In addition, several reports believe that enhanced autophagy can reduce APAP‐induced liver toxicity by blocking oxidative stress.^60^ However, the relationship between PB2, CS‐induced liver injury and autophagy has not yet been researched. Our results show that PB2 treatment induces autophagy by increasing protein levels of Beclin‐1 and LC3, whereas CS reduces these levels. Importantly, the PI3K/Akt/mTOR signalling is crucial in the initial stage of autophagosome formation.^61^ Initial activation of PI3K under stress conditions may increase free radicals in the vicinity of mitochondria. Proanthocyanidins induce autophagy because of the inhibitory effect of PI3K/AKT/mTOR in HepG2 cells.^62^ Therefore, it is necessary to explore the effect of PB2 on PI3K/AKT/mTOR signalling pathway. In the current study, we found that PB2 attenuated expression of PI3K, p‐AKT and mTOR, which is conducive to the activation of autophagy, indicating that PB2‐induced autophagy may be dependent on the PI3K/AKT/mTOR signalling pathway.

The precise target of PB2 was further investigated in the process of liver injury, and the Hh pathway attracted our attention. The Hh pathway plays an important role during various types of liver injury, such as fibrosis, inflammation‐related injury and carcinogenesis.^63^, ^64^ Emerging data show that Hh is a key regulator of adaptive and maladaptive responses to liver injury.^30^ The severity of liver fibrosis parallels the level of Hh activity in patients with chronic liver diseases.^65^ A recent study revealed that Hh signalling regulated hepatic inflammation in mice with non‐alcoholic fatty liver disease.^66^ Shh is the most studied ligand of the Hh signalling pathway and can interact with the receptor Patched in liver fibrosis and liver cancer cells.^67^ Patched eliminates the inhibitory effect on Smo, thereby promoting activation of transcription factor Gli1 and nuclear translocation, resulting in expression of Shh‐target genes, such as Smo and Gli1.^68^ We wanted to understand the interaction between PB2 and Shh signalling pathway in the liver under CS. Therefore, we used the Smo inhibitor cyclopamine to determine the role of PB2 in the Hh pathway in CS‐induced liver injury. PB2 exerted almost the same inhibitory effect on the Hh pathway as cyclopamine did. These findings primarily indicate that the Hh pathway is the target of PB2 in liver injury. We studied the relationship between Shh and autophagy activation and found that the Smo inhibitor cyclopamine increased activation of autophagy, indicating that addition of cyclopamine promotes the compensatory effect of autophagy activation. Taken together, we suggested that PB2‐mediated autophagy activation and Shh signalling inhibition improve liver injury caused by CS and that Shh signalling inhibition may enhance autophagy activation compensation.

**FIGURE 8:**
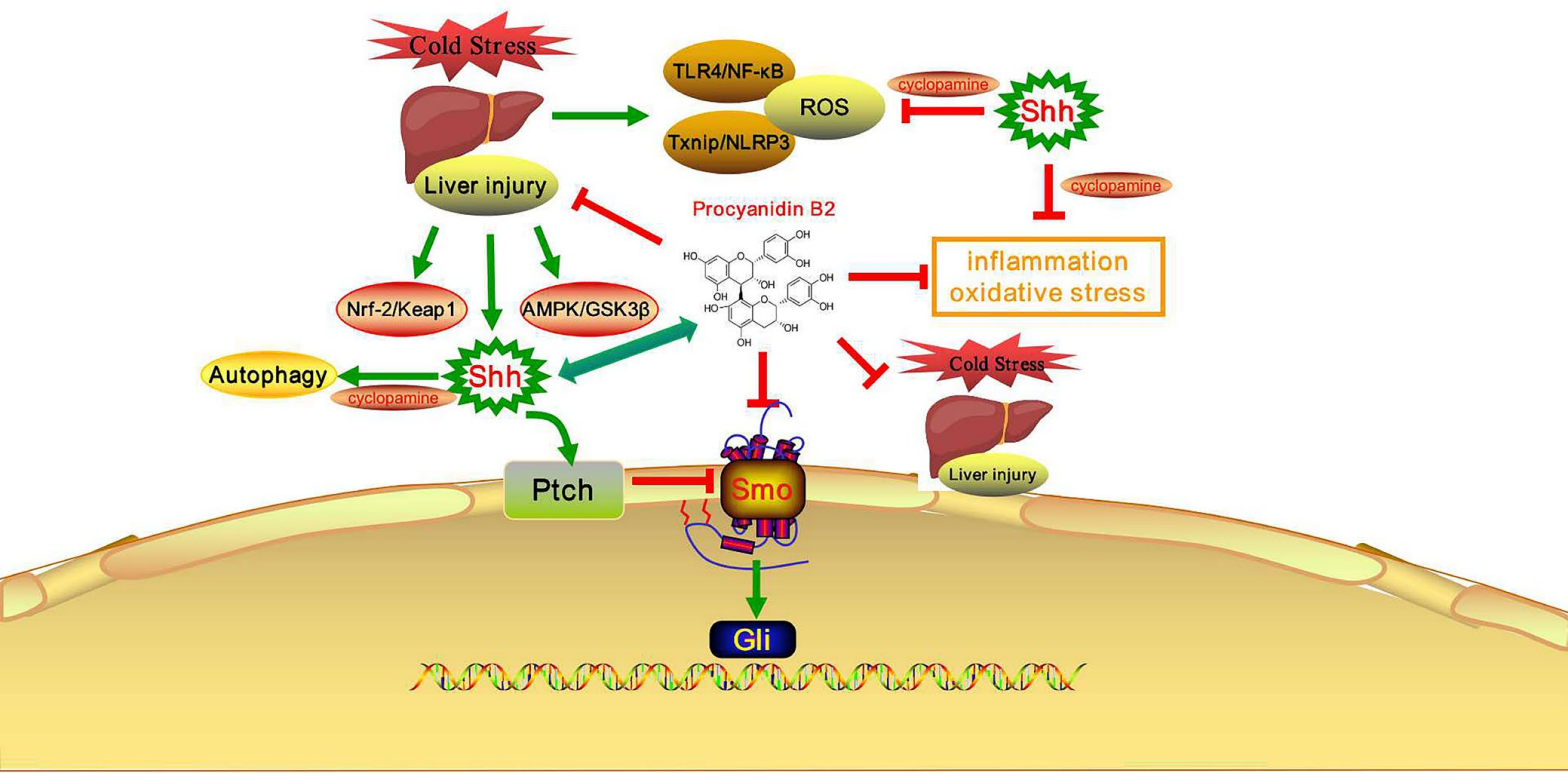
Scheme of the protective effects of PB2 on CS‐induced liver injury. A, Direct effects. PB2 induces autophagy activation and inhibits the Shh signalling pathway to alleviate inflammation and oxidative stress through inhibition of Txnip/NLRP3 as well as TLR4/NF‐κB, and activation of Nrf2/Keap1 and AMPK/GSK3β signalling pathways, which improves CS‐induced liver injury. B, Compensatory effects. Inhibition of the Shh signalling pathway may promote the compensatory effect of autophagy activation, thereby reducing the sensitivity of mice to CS. Inhibits the Shh signalling pathway and enhances PB2‐induced autophagy activation, thereby enhancing the ability of PB2 to protect liver injury in mice

In conclusion, we confirmed that PB2 can induce autophagy and inhibit the Shh signalling pathway to alleviate inflammation and oxidative stress by inhibiting Txnip/NLRP3 and TLR4/NF‐κB, and activating the Nrf2/Keap1 and AMPK/GSK3β signalling pathways, which improves CS‐induced liver injury (Figure 8). Additionally, inhibiting the Shh signalling pathway may promote the compensatory effect of autophagy activation, thereby reducing the sensitivity of mice to CS, inhibits the Shh signalling pathway and enhances PB2‐induced autophagy activation, thereby enhancing the ability of PB2 to protect liver injury in mice. Overall, the study provides new insights into the functional mechanism of PB2 and the inhibition of the Shh signalling pathway to protect the liver from inflammation and oxidative stress during CS‐induced liver injury.

## CONFLICTS OF INTEREST

The authors declare that there is no conflict of interest.

## AUTHOR CONTRIBUTIONS

**Li Ma:** Data curation (lead); Formal analysis (lead); Investigation (lead); Methodology (lead); Software (lead); Writing‐original draft (lead); Writing‐review & editing (lead). **Chengxu Li:** Formal analysis (equal). **Shuai Lian:** Methodology (equal); Writing‐original draft (equal). **Bin Xu:** Data curation (equal). **Hongming Lv:** Methodology (equal). **Yanzhi Liu:** Data curation (equal). **Jingjing Lu:** Methodology (equal). **Hong Ji:** Formal analysis (equal); Writing‐original draft (equal). **Shize Li:** Formal analysis (equal). **Jingru Guo:** Formal analysis (equal); Writing‐original draft (equal). **Huanmin Yang:** Formal analysis (equal); Funding acquisition (equal); Resources (equal); Writing‐original draft (equal).

## Data Availability

All data generated or analysed during this study are included in this article.
